# Research on the Depth Image Reconstruction Algorithm Using the Two-Dimensional Kaniadakis Entropy Threshold

**DOI:** 10.3390/s24185950

**Published:** 2024-09-13

**Authors:** Xianhui Yang, Jianfeng Sun, Le Ma, Xin Zhou, Wei Lu, Sining Li

**Affiliations:** 1 National Key Laboratory of Laser Spatial Information, Institute of Opto-Electronic, Harbin Institute of Technology, Harbin 150001, China; yangxianhui2019@163.com (X.Y.); hit_ml@126.com (L.M.); zx2021@hit.edu.cn (X.Z.); hit_luwei@163.com (W.L.); lisining@hit.edu.cn (S.L.); 2 Zhengzhou Research Institute, Harbin Institute of Technology, Zhengzhou 450000, China; 3 Research Center for Space Optical Engineering, Harbin Institute of Technology, Harbin 150001, China

**Keywords:** Gm-APD LiDAR, two-dimensional histogram, point cloud, Kaniadakis entropy

## Abstract

The photon-counting light laser detection and ranging (LiDAR), especially the Geiger mode avalanche photon diode (Gm-APD) LiDAR, can obtain three-dimensional images of the scene, with the characteristics of single-photon sensitivity, but the background noise limits the imaging quality of the laser radar. In order to solve this problem, a depth image estimation method based on a two-dimensional (2D) Kaniadakis entropy thresholding method is proposed which transforms a weak signal extraction problem into a denoising problem for point cloud data. The characteristics of signal peak aggregation in the data and the spatio-temporal correlation features between target image elements in the point cloud-intensity data are exploited. Through adequate simulations and outdoor target-imaging experiments under different signal-to-background ratios (SBRs), the effectiveness of the method under low signal-to-background ratio conditions is demonstrated. When the SBR is 0.025, the proposed method reaches a target recovery rate of 91.7%, which is better than the existing typical methods, such as the Peak-picking method, Cross-Correlation method, and the sparse Poisson intensity reconstruction algorithm (SPIRAL), which achieve a target recovery rate of 15.7%, 7.0%, and 18.4%, respectively. Additionally, comparing with the SPIRAL, the reconstruction recovery ratio is improved by 73.3%. The proposed method greatly improves the integrity of the target under high-background-noise environments and finally provides a basis for feature extraction and target recognition.

## 1. Introduction

Photon-counting LiDAR with three-dimensional imaging capability is widely used in autonomous driving [1], surveillance and defense [2], and military guidance [3]. Gm-APD LiDAR, as a new technology with single-photon sensitivity, has been a popular research topic in recent years and has been extensively studied by many research groups [4,5,6,7]. However, when Gm-APD receives an echo, the output signals are just 0 and 1, and it is impossible to distinguish avalanche events generated by signals or noise. Therefore, it is important to use algorithms to separate signal and noise photons in data processing [8,9]. As for long-range targets with low reflectivity [6,10] or power-constrained systems [11,12], the number of signal photons reflected from the target is small as compared to the number of noise photons, resulting in a signal that is very easily submerged in noise. Hence, it has become a great challenge to reconstruct the 3D structure of the target quickly and accurately under the high false alarm rate condition of Gm-APD LiDAR.

The echo photon count of the Gm-APD detector conforms to a Poisson distribution [13,14], and the signal cannot be estimated from a single frame of data, so it is necessary for accumulating multiple frames of data when reconstructing the target scene. The trigger events caused by the reflected laser from the target cluster in the histogram to form a signal peak whose amplitude reflects the reflectivity of the target surface, and, on the contrary, trigger events caused by the background light or the dark counts of the detector are usually uniformly distributed in the histogram. The peak method [15] is the simplest method to obtain the distance information, but the accuracy is reduced due to the fact that the method only compares the counts in each time bin without taking into account the influence of the waveform. On the basis of the peak method, Gaussian curve fitting and wavelet transform methods are used to improve the detection accuracy [16]. Researchers have used cumulative techniques for noise suppression, and the distance image of the target is obtained from using histogram information with mutual correlation methods or maximum likelihood estimation [17,18], which is an algorithm based on the mutual correlation of the echo signal with the instrument response function (IRF) to obtain the time-delayed information of the echoes. However, affected by the intensity of the background and signal light, such methods cannot accurately estimate the signal location in some extreme cases where the signal light is very close to the background light and the SBR is lower than 0.08 [10].

There are many emerging computational imaging techniques that are able to recover images with only a few photons. Kirmani et al. [13] designed a first-photon imaging system [13] to obtain single-pixel distance information using only the first echo photon of each pixel and exploiting the spatial correlation of neighboring pixels. Subsequently, Shin et al. proposed a sparse regularization reconstruction algorithm [18] based on first-photon imaging to achieve 3D imaging with an average of ~1 photon per pixel, combining with related computational imaging methods, and they further proposed a reconstruction algorithm based on the joint subspace model [19]. In 2016, Altmann et al. [20] utilized the Bayesian estimation method in image reconstruction, proposing a framework based on hierarchical Bayesian model reconstruction. In 2017, Halimi et al. used the hierarchical Bayesian approach [21] to reconstruct the scene in strongly attenuated and noisy underwater environments with high accuracy and proposed a reconstruction algorithm [22] based on nonlocal analysis for imaging in smoke environments to achieve image reconstruction in cases of SBRs of less than 1. In 2017, Rapp [23] proposed an unmixing algorithm that can accurately reconstruct the scene of an SBR as low as 0.04. Peng et al. [24] designed a hybrid penalty method based on wavelet *l*_1_-norm and total variation for the first-photon imaging system, which balanced the denoising effect with the image details. Hua et al. [25] designed a first signal photon unit (FSPU) method to rapidly reconstruct depth images from sparse signal photon counts with strong noise robustness, which processed the point-by-point reconstruction results by median filtering and TV regularization. Ma et al. [26] used the spatial correlation of echo signals to establish a reliable target image, used the consistency of depth and intensity images to establish a reliable initial value, and, finally, reconstructed the image by a Markov random field (MRF) and correlation, which resulted in a target recovery of up to 19.40% when the SBR was 0.0055.

However, for typical algorithms in the past, the Peak-picking method extracts the maximum peak directly, the Cross-Correlation method extracts the maximum peak position by matching the filter, and the sparse Poisson intensity reconstruction algorithm [10] obtains the peak position by a 3D spatio-temporal matrix to solve reflectivity and depth simultaneously. However, in the algorithms, only one peak signal is extracted, and there is a high probability that the extracted peak is noise. In particular cases where the background noise is strong or the SBR is relatively low, the signal is submerged in the noise and the correct signal cannot be extracted. Additionally, in practical applications, when the accumulation time is not long enough, it is impossible to accumulate enough signal photons to extract the real signal when just one peak is extracted, resulting in a poor reconstruction effect.

To solve this problem, we propose a reconstruction method based on 2D Kaniadakis thresholding. First, the multiple distance values are extracted from the histogram of each image element to construct 3D point cloud data; then, the threshold range of the target waveform, extracted from the histogram of the full domain using the derivative method, is used for preliminary noise filtering of the point cloud-intensity data. Finally, based on the target spatial correlation, the thresholds of the point cloud-intensity data are obtained using the 2D Kaniadakis method, and the point cloud-intensity data are filtered with noise to obtain the depth image.

## 2. Proposed Method

**Figure 1:**
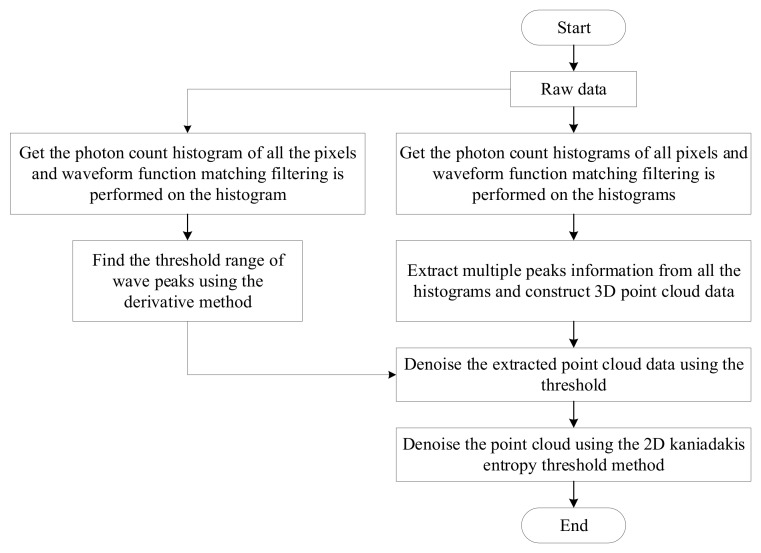
Flowchart of the proposed algorithm.

Figure 1 shows the flowchart of the image reconstruction algorithm, which seeks the threshold value based on the spatio-temporal characteristics of the data. First, we smooth the histogram of all pixels using a matched filtering algorithm and estimate the distance information of a number of peaks and the intensity information corresponding thereto before putting the distance information and the intensity information into 3D space to construct the point cloud-intensity data. Second, the histogram of the full domain pixels is counted, smoothed using a matched filtering algorithm, and the derivative method is used to find the threshold range of the wave peaks in the histogram; the point cloud-intensity data are then initially filtered for noise. Finally, based on the target spatial correlation, the two-dimensional histograms of the point cloud density and the mean intensity of the neighborhood are counted, the thresholds of the point cloud density and the mean intensity are obtained using the 2D Kaniadakis method, and the point cloud-intensity data are filtered further with noise to obtain the depth image.

### 2.1. Construction of Point Cloud-Intensity Data

During the GM-APD detector detection, the photon detection event is a non-uniform Poisson process. Based on the time resolution of the detector’s time converter, the time-delayed gate is evenly divided into time bins with the same time interval. According to the Poisson distribution model, the trigger probability of the *k*th time bin is tantamount to the product of the untriggered probability of the previous *k* − 1-time bins and the trigger probability of the *k*th time bin. The trigger probability of the *k*th time bin is [27]:

(1) $$\begin{array}{cc} P_{k}=\exp\left(-\sum_{l=0}^{k-1}\left(S_{l}+N_{l}\right)\right)[1-\exp(-S_{k}-N_{k})] & k=1, \end{array}2,3\ldots K,$$

where

(2) $$S_{k}+N_{k}=\int_{(k-1)\cdot t_{bin}}^{k\cdot t_{bin}}r(t)dt,$$

*S_k_* + *N_k_* denotes the number of photons reaching this detector at the *k*th time bin. Here, *t_bin_* is the duration of each time bin and *r*(*t*) denotes the photon rate function, which can be expressed as:

(3) $$r(t)=\eta (n_{1}(t)+n_{2}(t))+n_{d}(t),$$

where *η* is the quantum efficiency, *n*_1_(*t*) and *n*_2_(*t*) are the rate functions of noise and signal echo, respectively, and *n*_1_(*t*) is considered to be a constant, *n*_2_(*t*) is related to the intensity of the signal, and *n_d_*(*t*) is the dark count.

**Figure 2:**
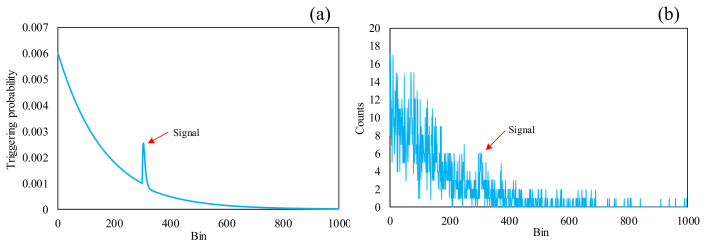
(**a**) Distribution of the probability density of the signal and noise when the signal position is at the 300th time bin. (**b**) Counting histogram of 0.1 s data with signal and noise.

Figure 2a shows the simulated trigger probability curve of the Gm-APD with a delay gate width of 1000 time bins, the noise is uniformly distributed within the time bins, and the signal position is at the 300th time bin. The bin means the value within the delay gate of the LiDAR detector.

**Figure 3:**
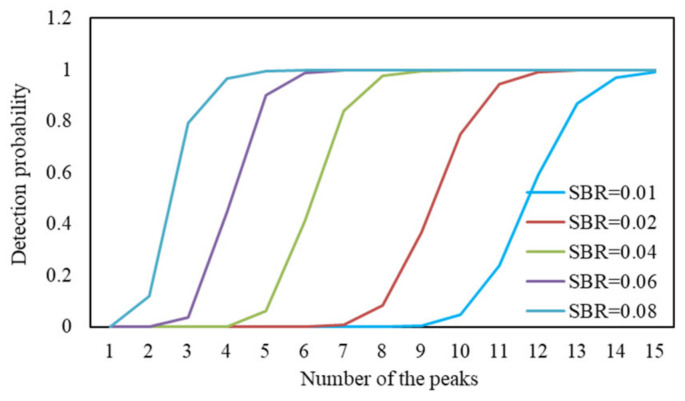
Signal detection rate for extracting different numbers of wave peaks under different SBRs.

As shown in Figure 3, the detection rate curves of the echo signals are shown for different SBRs when a different number of peaks are extracted in the histogram.

As can be seen in Figure 3, the detection probability of the signal is 0 when only 1 wave peak is extracted at a very low SBR. The lower the SBR, the more wave peaks need to be extracted to ensure that the signal is not missed. When the SBR is 0.01, at least 15 waveforms need to be extracted to ensure that the signal is not missed. Therefore, extracting multiple peaks in each histogram can improve the detection rate of the signal and reduce the effect of background noise.

The histogram of each pixel is counted, and, in order to reduce the influence of abnormal peaks, the histogram is convolved using a Gaussian function to obtain a smoothed histogram [28].

The locations of multiple peaks are extracted from the smoothed histogram according to their intensities, and the depth and intensity values correspondent to the peaks are placed into 3D space to obtain 3D point cloud-intensity data.

### 2.2. Automatic Thresholding by Derivative Method

As can be seen in Figure 2, when the background noise of the scene is particularly high, the peaks in the first half of the delay gate are larger than the peaks in the second half of the delay gate, resulting in the point cloud data extracted in Section 2.1 being aggregated in front of the delay gate; this has a significant impact on the subsequent point cloud denoising algorithm, so thresholds need to be extracted to remove the noise at the open door of the delay gate.

The histogram of the detector’s all-pixel multi-frame data is counted, and the range of the peaks in the histogram is estimated using the derivative method [29] to find the minimum and maximum threshold ranges (*T_l_*, *T_h_*) for the peaks. The point cloud-intensity data in Section 2.1 is processed by filtering out the points whose distance values are not within the threshold range (*T_l_*, *T_h_*) to obtain the point cloud-intensity data of the preliminary filtered noise.

### 2.3. Two-Dimensional Kaniadakis Entropy Thresholding Method

In order to filter out the remaining noisy points in the point cloud-intensity data obtained in Section 2.2, a 2D Kaniadakis entropy thresholding algorithm is used to obtain the final point cloud image based on the similarity of the target structure and the spatio-temporal features of the data. The points in the target area are clustered together, while the noise points are disordered and random, and the intensity values of the points on the target are larger than those of the noise points, so the two features selected are the number of points and the average intensity value of the points in the neighborhood box.

If *f*(*x*, *y*, *z*) denotes the intensity value at the point (*x*, *y*, *z*) and the dimensional size of the 3D space is *M* × *N* × *G* where *x*∈[1, 2, …, *M*], *y*∈[1, 2, …, *N*], *z*∈[1, 2, …, *G*], *f*(*x*, *y*, *z*)∈*F* = [0, 1, …, *L*], *M* and *N* are the maximum values of horizontal and vertical pixels in 3D space, respectively, and *G* is the maximum depth value in 3D space. In the experiment, *G* is the number of time bins in the detector delay gate and *L* is the maximum intensity value of the 3D data.

For the 3D data, *g*(*x*, *y*, *z*) is used to denote the number of points in the neighborhood of coordinates (*x*, *y*, *z*) with a neighborhood box size of *X* × *Y* × *Z*. *X*, *Y*, and *Z* are the width, height, and length of the neighborhood box, respectively, which is usually odd. *g*(*x*, *y*, *z*) can be computed according to the following equation [30,31], and *L*_1_ is the maximum value of *g*(*x*, *y*, *z*).

(4) $$g(x,y,z)=\sum_{m=-(X-1)/2}^{(X-1)/2}\sum_{n=-(Y-1)/2}^{(Y-1)/2}\sum_{o=-(Z-1)/2}^{(Z-1)/2}\frac{f(x+m,y+n,z+o)}{f(x+m,y+n,z+o)},$$

*h*(*x*, *y*, *z*) denotes the average intensity of the points in the neighborhood of pixel (*x*, *y*, *z*) with a neighborhood size of *X* × *Y* × *Z*. *h*(*x*, *y*, *z*) can be calculated according to the following equation, and *L*_2_ is the maximum value of *h*(*x*, *y*, *z*).

(5) $$h(x,y,z)=\left\lfloor \frac{1}{g(x,y,z)}\sum_{m=-(X-1)/2}^{(X-1)/2}\sum_{n=-(Y-1)/2}^{(Y-1)/2}\sum_{o=-(Z-1)/2}^{(Z-1)/2}f(x+m,y+n,z+o)\right\rfloor ,$$

The binary group consisting of *g*(*x*, *y*, *z*) and *h*(*x*, *y*, *z*) is denoted as (*i*, *j*). The value of the 2D histogram is denoted by *P_ij_*, which represents the frequency of (*i*, *j*), and *P_ij_* can be calculated using the following equation.

(6) $$P_{ij}=\frac{c_{ij}}{M\times N\times G},$$

where *c_ij_* is the number of occurrences of (*i*, *j*), 0 ≤ *i* ≤ *L*_1_, 0 ≤ *j* ≤ *L*_2_, $\sum_{i=0}^{L_{1}}\sum_{j=0}^{L_{2}}p_{ij}=1$.

The optimal threshold is chosen by maximizing the 2D Kaniadakis entropy [32], and the segmented 3D data *ḟ*(*x*, *y*, *z*) can be obtained by the optimal threshold (s*, t*).

(7) $$\dot{f}(x,y,z)=\left\{\begin{array}{cc} nan & g(x,y,z)<s^{*},h(x,y,z)<t^{*}\\ f(x,y,z) & others \end{array}\right.,$$

The obtained 3D data are transformed into a 2D image by taking *x* and *y* as the image element horizontal and vertical coordinates, respectively, and *z* as the depth value of the coordinate (*x*, *y*).

## 3. Evaluation and Simulation

### 3.1. Evaluation

When the depth image is reconstructed by using different algorithms, it is necessary to use objective evaluation metrics to assess the performance of each algorithm. Here, we choose the recovery rate (*P_r_*) and root mean square error (*RMSE*) to evaluate the reconstruction results.

(8) $$P_{r}=\frac{n}{m_{truth}},$$

where *m_truth_* is the number of pixels of the target in the truth image and *n* is the total number of target pixels recovered using different algorithms. Additionally, *n* is the pixels number of the target that satisfies the distance threshold, and the satisfied pixels can be expressed by the following equation:

(9) $$ima_{ij}=\left\{\begin{array}{cc} 1 & \left|d_{ij}-d_{ij}^{truth}\right|<d_{r}\\ 0 & \left|d_{ij}-d_{ij}^{truth}\right|\geq d_{r} \end{array}\right.,$$

where *d_r_* is the laser pulse width, *d_ij_* is the value of the recovered depth image, $d_{ij}^{truth}$ is the value of the truth depth image, and *i* and *j* are the coordinates of the 2D image element. The *n* is the number of image pixels equal to 1 in the image.

(10) $$RMSE=\frac{{\left\|d^{truth}-d\right\|}_{2}}{e_{v}\cdot e_{h}},$$

where *e_h_* and *e_v_* are the maximum number of image elements in the horizontal and vertical directions of the image, respectively.

### 3.2. Simulation

**Figure 4:**
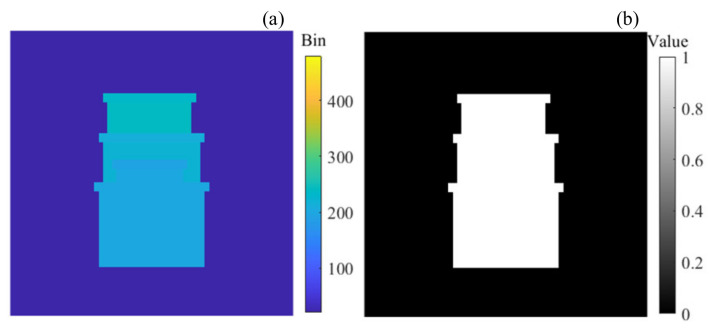
(**a**) The standard depth image. (**b**) The standard intensity image.

The advancement of the proposed method is tested using a simulation. The simulated data are obtained by using a model based on the Monte Carlo method, and the depth image results obtained by using different methods are evaluated using the recovery rate (*P_r_*) and the root mean square error (*RMSE*). A Gm-APD LiDAR system with a number of 64 × 64 pixels and a distance resolution of 0.15 m is simulated using the simulation model. The standard image of the simulation input is shown in Figure 4.

**Figure 5:**
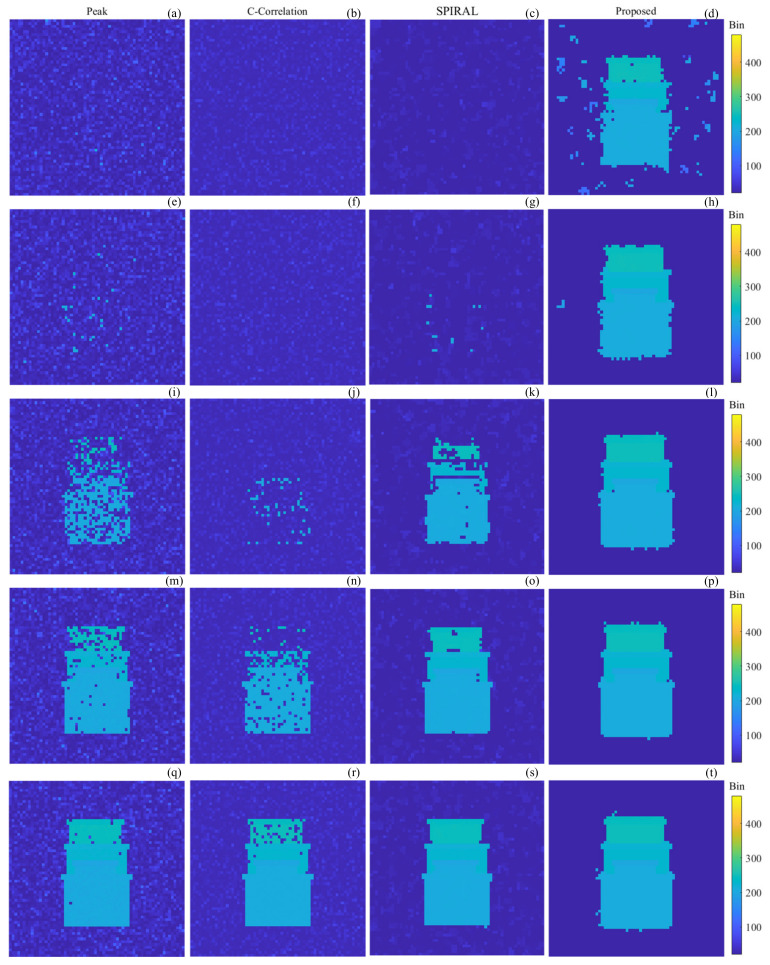
Depth images obtained using different methods of reconstruction. (**a**–**d**) SBR is 0.01; (**e**–**h**) SBR is 0.02; (**i**–**l**) SBR is 0.04; (**m**–**p**) SBR is 0.06; (**q**–**t**) SBR is 0.08; (**a**,**e**,**i**,**m**,**q**) Peak-picking method; (**b**,**f**,**j**,**n**,**r**) Cross-Correlation method; (**c**,**g**,**k**,**o**,**s**) SPIRAL method; (**d**,**h**,**l**,**p**,**t**) proposed method.

The number of background noise photons is set to be 6, which is uniformly distributed in the delay gate, and the number of signal photons is set to be 0.06, 0.12, 0.24, 0.36, and 0.48, separately, to obtain the simulation data with SBRs of 0.01, 0.02, 0.04, 0.06, and 0.08, respectively. The simulation data are processed using Peak-picking, Cross-Correlation, SPIRAL [10], and our proposed method, respectively, to obtain the depth image, as shown in Figure 5.

From Figure 5, it can be seen that when the SNR is less than 0.02, the Peak-picking method, Cross-Correlation method, and SPIRAL method can hardly reconstruct the target or only a few pixels are reconstructed. The Peak-picking method and Cross-Correlation method are able to reconstruct the target completely when the SBR increases to 0.08, and the SPIRAL method is able to reconstruct the target completely when the SBR increases to 0.06. However, as for the proposed method, when the SBR is 0.01, most of the pixels of the target can be reconstructed, but there is some noise in the image and the edges of the target are incomplete, being both overestimated and not reconstructed. Additionally, when the SBR is greater than 0.02, there is only the complete target in the reconstructed result and almost no noise. However, the images of the proposed method are extended by one pixel outward than the standard image; this is because our proposed method uses a neighborhood and the neighborhood size is 7 × 7 × 15, which leads to the noisy image elements at the edges being treated as targets when thresholding.

The recovery rate and RMSE of the depth image are used to evaluate the quality of the image to verify the effectiveness of the proposed algorithm. The recovery rate only evaluates the percentage of image pixels reconstructed by the algorithm, but it does not take into account the overestimation. Additionally, the RMSE can evaluate the difference with the standard image, including the overestimation and the denoising.

The recovery rate and root mean square error of the depth image in Figure 5 are calculated as shown in Table 1.

From Table 1, it can be seen that the recovery rate of the Peak-picking method, Cross-Correlation method, and SPIRAL method grow when the SBR grows; the recovery rate of Peak-picking method and SPIRAL method are close to 1 when the SBR is 0.06; when the SBR is 0.08, the recovery rate of the Cross-Correlation method is close to 1; when the SBR is 0.01, the recovery rate of the proposed method is 97.7%; and when the SBR is bigger than 0.01, the recovery rate of the proposed method is always close to or equal to 1.

The RMSE of the proposed method is stable near 0.6 when the SBR is 0.01, 0.02, 0.04, 0.06, and 0.08. As the SBR increases, the RMSE of the Peak-picking method, Cross-Correlation method, and SPIRAL method decreases; when the SBR are 0.06 and 0.08, the RMSE of the SPIRAL method is smaller than the proposed method; when the SBR is 0.08, the RMSE of the Peak- picking method and Cross-Correlation method is slightly smaller than the proposed method.

**Figure 6:**
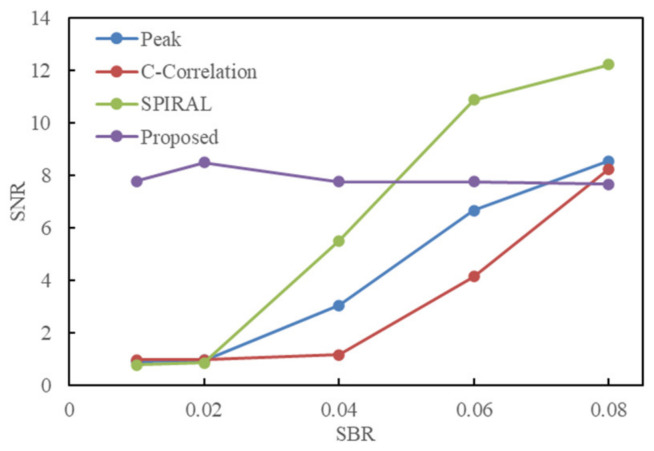
The relationship between the SBR of the scene and the SNR of the reconstructed image using different methods.

Calculate the SNR of the reconstructed image using different methods and obtain the relationship between the SNR of the image and the SNR of the scene, as shown in Figure 6.

It can be seen that, when the SBR of the scene increases from 0.01 to 0.08, the SNR of the reconstructed results of the Peak-picking method, Cross-Correlation method, and SPIRAL method increases. When the SBR is 0.06, the SNR of the reconstructed image by the SPIRAL method is larger than that by the proposed method. When the SBR of the scene is between 0.01 and 0.08, the SNR of the reconstructed result by the proposed method is almost stable between 7.5 and 8.5, which is almost considered to be a constant. When the SBR of the scene is 0.01 or lower, the echo signal becomes weaker, and it is necessary to increase the accumulation time of the data to be able to extract a strong enough echo signal to ensure the extraction of the echo signal when extracting multiple waveforms, which in fact has a certain limitation on the range of application scenarios of the proposed algorithm.

## 4. Experiments and Discussion

In order to verify the effectiveness of the proposed method, two sets of outfield experiments are performed using a Gm-APD detector. In the experiments, scene data with different SBRs are obtained by using the detector to image the target at different times and by setting different laser energies, and the intensity of the background light varies at different times. The detector image pixel number is 64 × 64, the laser pulse width is 15 ns, and the detector frequency is also 20,000 Hz.

**Figure 7:**
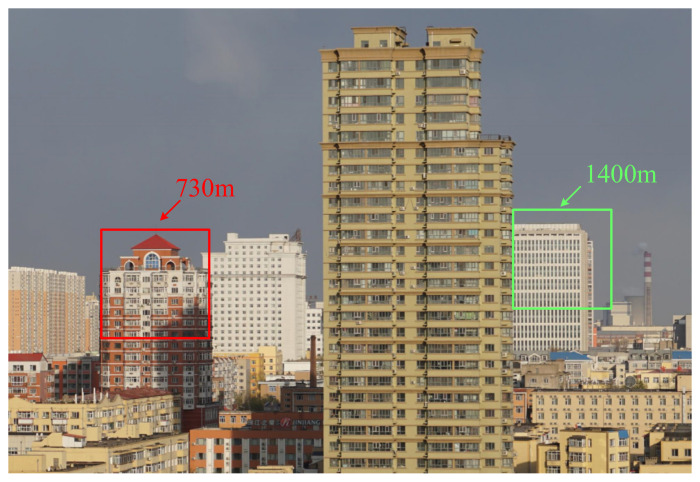
The photos of the experimental targets.

We collect data of different scenes and calculate the SBRs. When calculating the SBR of the scenes, we count the histogram of all pixels of the target and count the number of echo photons per time bin within the delay gate. Then, the sum of the signal and noise echo photons is calculated, and the number of echo photons of the signal is divided by the number of echo photons of the noise to obtain the SBR of the scene. Target 1 is a building with a distance of 730 m, and the SBRs of the experimental scenes are 0.078, 0.053, and 0.031, respectively. Target 2 is a building with a distance of 1400 m, and the SBRs of the experimental scenes are 0.031 and 0.025, respectively. As shown in Figure 7, the building in the red box is target 1 and the red box is the detector field of view; the one in the green box is target 2 and the green box is the detector field of view.

**Figure 8:**
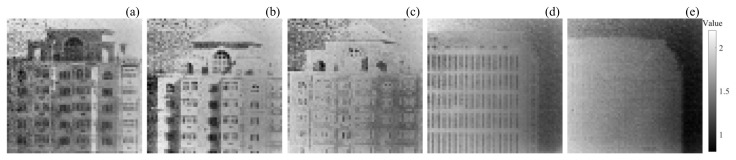
The number of echo photons/pixel of the scenes. (**a**) Building at a distance of 730 m with an SBR of 0.078. (**b**) Building at a distance of 730 m with an SBR of 0.053. (**c**) Building at a distance of 730 m with an SBR of 0.031. (**d**) Building at a distance of 1400 m with an SBR of 0.031. (**e**) Building at a distance of 1400 m with an SBR of 0.025.

The number of echo photons/pixels in the scenes is shown in Figure 8. The intensity of the gray color on the right side of the image indicates the number of photons in the image. The maximum value of the 730 m building with SBRs of 0.078, 0.053, 0.031 are about 2.1, 2.1, 2.0, and the maximum value of the 1400 m building with SBRs of 0.031, 0.025 is about 2,1, 2.0.

In our proposed method, *M*, *N* and *G* are 64, 64, and 1000, respectively. The neighborhood size is *X* × *Y* × *Z*, where *X* = 7, *Y* = 7, *Z* = 15. Among them, the choice of *X* and *Y* is related to the size of the scene. In our experimental scene, the image quality is better when the size of *X* and *Y* are 7 and 7 respectively, which does not result in filtering out too many target pixels and retaining more noise. *Z* is equal to the laser pulse width. For a two-dimensional Kaniadakis entropy threshold in the proposed method, the larger the parameter *κ*, the larger the threshold obtained, and, for our experimental scene, the method performs well when the parameter *κ* is 0.1.

**Figure 9:**
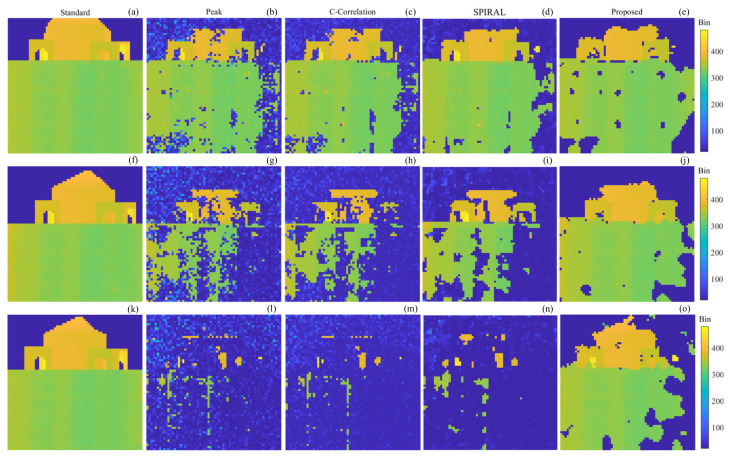
Truth depth images and depth images obtained using different methods of the 730 m building. (**a**–**e**) SBR is 0.078; (**f**–**j**) SBR is 0.053; (**k**–**o**) SBR is 0.031; (**a**,**f**,**k**) truth depth image; (**b**,**g**,**l**) Peak-picking method; (**c**,**h**,**m**) Cross-Correlation method; (**d**,**i**,**n**) SPIRAL method; (**e**,**j**,**o**) proposed method.

As shown in Figure 9, the depth images of the building at a distance of 730 m with SBRs of 0.078, 0.053, 0.031, respectively, are shown, along with the truth depth images and the depth images obtained using Peak-picking, Cross-Correlation, SPIRAL, and our proposed method, respectively. Among them, the truth images are obtained with 1 s data using the Peak-picking method, and some pixels are processed manually. The depth images of the other methods are obtained using 0.1 s data.

As can be seen in Figure 9, the number of pixels of the depth image reconstructed by each method is roughly the same for the scene with an SBR of 0.078. When the SBR decreases to 0.053, the number of pixels of the depth image reconstructed by each method decreases, but the proposed method has the highest number of reconstructed pixels. When the SBR decreases to 0.031, the number of image pixels of the target reconstructed by the Peak-picking method, Cross-Correlation method, and SPIRAL method decreases drastically, and the target is almost invisible, while the number of image pixels in the reconstructed target by the proposed method decreases but the target can still be distinguished.

**Figure 10:**
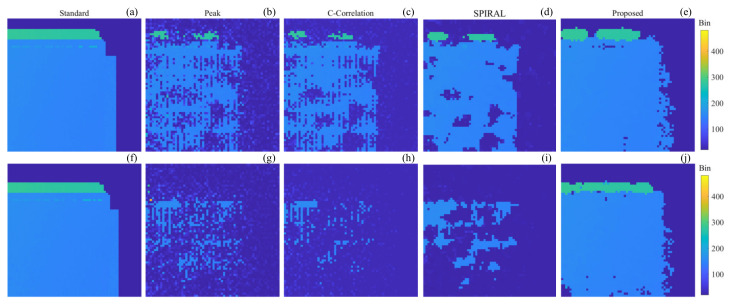
Truth depth images and depth images obtained using different methods of the 1400 m building. (**a**–**e**) SBR is 0.031; (**f**–**j**) SBR is 0.025; (**a**,**f**) truth depth image; (**b**,**g**) Peak-picking method; (**c**,**h**) Cross-Correlation method; (**d**,**i**) SPIRAL method; (**e**,**j**) proposed method.

As shown in Figure 10, the depth images of the building at a distance of 1400 m with SBRs of 0.031 and 0.025, respectively, are shown, along with the truth depth images and the depth images obtained using Peak-picking, Cross-Correlation, SPIRAL, and our proposed method, respectively. Among them, the truth images are obtained with 1 s data using the Peak-picking method, and some pixels are processed manually. The depth images of the other methods are obtained using 0.1 s data.

As can be seen in Figure 10, for the scene with an SBR of 0.031, the Peak-picking method and Cross-Correlation method reconstruct the target with approximately the same number of target pixels, and the reconstruction results are the worst; the SPIRAL method reconstructs the target better than the Peak-picking method and Cross-Correlation method, but there are some pixels of the target that have not been reconstructed; and, in contrast, the proposed method has the best reconstruction results, which reconstructs the target almost completely. When the SNR decreases to 0.025, the results of the Peak-picking method, Cross-Correlation method, and SPIRAL method all become worse, the number of reconstructed pixels of the target decreases, and the 3D structure of the target cannot be visualized, whereas the reconstruction results of the proposed method almost remain the same and the complete target can still be reconstructed.

The recovery rate and root mean square error of the depth image in Figure 9 and Figure 10 are calculated as shown in Table 2.

From Table 2, it can be seen that the proposed method has the highest recovery rate of the reconstructed images and has the most complete target under different SBR conditions. For target 1, the RMSE of the Peak-picking method, Cross-Correlation method, and SPIRAL method increases gradually with the decrease in SBR of the scenes from 2.08 to 4.33, while the RMSE of the proposed method is almost stable near 2. When the SBR is 0.031, the recovery rates of the Peak-picking method, Cross-Correlation method, and SPIRAL method are below 7%, while the proposed method achieves 78.3%, which improves the recovery rate by at least 71.3%.

For target 2, when the SBRs are 0.031 and 0.025, the RMSEs of the proposed method are the smallest, which are 0.64 and 0.59, respectively, while the minimum value of the RMSE of the Peak-picking method, Cross-Correlation method, and SPIRAL method is 1.12. The proposed method also has the largest recovery rates, which are above 90% with 93.6% and 91.7% for both SBRs of 0.031 and 0.025, respectively, while the highest recovery rates among the three methods of the Peak-picking method, Cross-Correlation method, and SPIRAL method are achieved by SPIRAL, which are 69.5% and 18.4%, respectively. Additionally, the proposed method improves the recovery rate by 73.3% over SPIRAL with an SBR of 0.025.

In the proposed method, when the SBRs of target 2 are 0.031 and 0.025, they have higher recovery rates and smaller RMSEs than target 1, with SBRs of 0.031, 0.053, and 0.078. This is because the 3D structure of target 2 is simpler than target 1, and the surface material of target 2 is similar, with roughly the same reflectivity, which leads to a good reconstruction result, although the SBR of target 2 is lower than target 1. Meanwhile, the Target 1 has a complex 3D structure and its materials have different reflectivity, and this indicates that the laser signals received by the detector are uneven in intensity and the intensity image is not uniform, which in turn results in some pixels with smaller intensity values being filtered out as noise during denoising using the derived thresholds.

**Figure 11:**
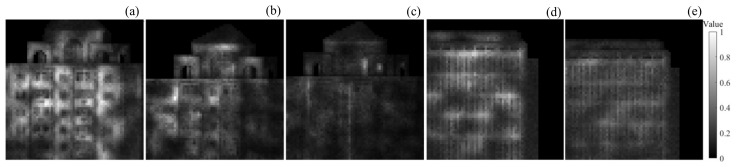
Intensity images with different signal-to-background ratios. (**a**) A 730 m building intensity image with an SBR of 0.078; (**b**) a 730 m building intensity image with an SBR of 0.053; (**c**) a 730 m building intensity image with an SBR of 0.031; (**d**) a 1400 m building intensity image with an SBR of 0.031; (**e**) a 1400 m building intensity image with an SBR of 0.025.

As shown in Figure 11, there are normalized intensity images of target 1 and target 2 under different SBRs. They are obtained by extracting the intensity values of the corresponding pixels in the histogram based on the values of the truth depth images and then normalizing them uniformly. The intensity of the image is not only related to the distance but also to the background light, signal light intensity, target angle, and reflectivity. Additionally, there is a high probability that the intensity of the targets that are farther away is stronger than targets that are closer.

From Figure 11a–e, it can be seen that the intensity of target 2 is more uniform than target 1. As the SBR decreases, the signal echo becomes weaker and weaker with respect to the noise, the intensity values in the intensity image decrease, and the Peak-picking method, Cross-Correlation method, and SPIRAL method fail to extract the correct distance values of the image pixels with low intensity values in the intensity image of Figure 11, which are considered to be noises. However, the proposed method recovers the majority of the image pixels with low intensities and is able to reconstruct a more complete target under the same SBR, leaving only a small portion of image elements that failed to be reconstructed.

The mean and standard deviation of the target region, the reconstructed target region, and the unreconstructed target region using the proposed method from Figure 11 are shown in Table 3.

As can be seen from the table, when the SBR is 0.078 for a 700 m building, the mean and standard deviation of the target region are 0.3168 and 0.1680, respectively. When the SBR is 0.053, the mean and standard deviation of the target region are 0.2124 and 0.1213, respectively. When the SBR is 0.031, the mean and standard deviation of the target region are 0.1283 and 0.0610, respectively. When the SBR is 0.031 for a 1400 m building, the mean and standard deviation of the target region are 0.2674 and 0.1181, respectively. When the SBR is 0.025, the mean and standard deviation of the target region are 0.2300 and 0.0773, respectively. When the SBR decreases, the mean intensity of the total target decreases, as does the standard deviation.

It can be seen from the mean and standard deviation of target 1, with SBRs of 0.078 and 0.031, as well as target 2, that the larger the intensity mean and the smaller the standard deviation, the higher the recovery rate.

From the mean and standard deviation of target 1, with SBRs of 0.078 and 0.053 as well as the mean and standard deviation of target 1 with an SBR of 0.078 and target 2, it can be seen that, although the mean value of the intensity decreases, the recovery rate increases instead after the standard deviation decreases so that the recovery rate of the image is not only related to the mean value but also more related to the standard deviation of the intensities.

From the intensity mean values of the reconstructed and unreconstructed regions of the target 1 and the target 2, it can be seen that the smaller the intensity mean value of the reconstructed region, the smaller the intensity mean value of the unreconstructed region.

In summary, the recovery rate of the proposed algorithm is positively correlated with the intensity mean, negatively correlated with the intensity standard deviation, and is more affected by the intensity standard deviation.

## 5. Conclusions

In this paper, the spatio-temporal characteristics of signals from Gm-APD LiDAR are analyzed in depth, and the depth image estimation method based on a 2D histogram is proposed. Multiple peaks are extracted in the histogram of each pixel to ensure the detection rate of the signal. The threshold range, found by the derivative method, initially filters the 3D point cloud-intensity data constituted by the multiple peaks to reduce the data complexity. Thresholds for density and mean intensity features are automatically derived using the 2D Kaniadakis entropy threshold method, which further filters the point cloud-intensity data to obtain depth images. Sufficient simulations and outdoor target-imaging experiments show that the proposed method has better results than the existing typical methods, and the recovery rate of the target reaches 91.7% in a scene with an SBR of 0.025. The proposed method can achieve efficient imaging of targets under low SBR conditions with a stable target integrity and is suitable for large target depth imaging under strong noise interference.

However, the performance of the proposed method is not good enough when the target intensity image is not uniform and the pixel with low intensity cannot be reconstructed. Moreover, this method is not enough in regard to image detail processing, such as with image edges. For remote small targets in scenes featuring low SBRs in particular, the target may not be reconstructed due to insufficient detail processing ability. In the future, the imaging of complex structural targets with uneven intensity and remote small targets under low SBR conditions will be investigated to further improve the imaging performance of the Gm-APD LiDAR.

## Figures and Tables

**Table 1:** The evaluation of reconstruction results in different SBR.

*SBR*	Peak-Picking		Cross-Correlation		SPIRAL		Proposed	
	*Pr*	*RMSE*	*Pr*	*RMSE*	*Pr*	*RMSE*	*Pr*	*RMSE*
0.01	0.1%	1.44	0	1.43	0	1.46	97.7%	0.63
0.02	2.8%	1.43	0	1.43	1.6%	1.44	1	0.58
0.04	49.3%	1.12	6.1%	1.39	74.7%	0.84	99.9%	0.63
0.06	89.2%	0.73	65.9%	0.98	97.2%	0.44	1	0.63
0.08	98.8%	0.58	95.3%	0.60	99.7%	0.38	1	0.63

**Table 2:** The evaluation of reconstruction results of the two targets in different SBRs.

		Peak-Picking		Cross-Correlation		SPIRAL		Proposed	
	SBR	*Pr*	*RMSE*	*Pr*	*RMSE*	*Pr*	*RMSE*	*Pr*	*RMSE*
Target 1	0.078	73.8%	2.30	75.9%	2.24	81.4%	2.08	82.8%	2.21
	0.053	38.0%	3.34	35.7%	3.42	47.9%	3.18	83.6%	2.03
	0.031	6.0%	4.23	4.2%	4.26	6.5%	4.33	78.3%	2.25
Target 2	0.031	51.0%	1.26	53.9%	1.26	69.5%	1.12	93.6%	0.64
	0.025	15.7%	1.65	7.0%	1.69	18.4%	1.71	91.7%	0.59

**Table 3:** Means and standard deviations of different regions of the intensity image.

		Total Target		Reconstructed Region		Unreconstructed Region	
	SBR	*μ*_1_	*σ*_1_	*μ*_2_	*σ*_2_	*μ*_3_	*σ*_3_
Target 1	0.078	0.3168	0.1680	0.3518	0.1588	0.1386	1.7792
	0.053	0.2124	0.1213	0.2354	0.1165	0.0802	0.6262
	0.031	0.1283	0.0610	0.1441	0.0576	0.0652	0.4679
Target 2	0.031	0.2674	0.1181	0.2746	0.1163	0.1304	0.6099
	0.025	0.2300	0.0773	0.2375	0.0745	0.1283	0.3389

## Data Availability

The data presented in this study are available on request from the corresponding author due to privacy.
